# Magnetic Bistability for a Wider Bandwidth in Vibro-Impact Triboelectric Energy Harvesters

**DOI:** 10.3390/mi14051008

**Published:** 2023-05-07

**Authors:** Qais Qaseem, Alwathiqbellah Ibrahim

**Affiliations:** Department of Mechanical Engineering, The University of Texas at Tyler, 3900 University Blvd., Tyler, TX 75799, USA

**Keywords:** triboelectric, energy harvesting, bistable, vibro-impact, bandwidth, magnet

## Abstract

Mechanical energy from vibrations is widespread in the ambient environment. It may be harvested efficiently using triboelectric generators. Nevertheless, a harvester’s effectiveness is restricted because of the limited bandwidth. To this end, this paper presents a comprehensive theoretical and experimental investigation of a variable frequency energy harvester, which integrates a vibro-impact triboelectric-based harvester and magnetic nonlinearity to increase the operation bandwidth and improve the efficiency of conventional triboelectric harvesters. A cantilever beam with a tip magnet was aligned with another fixed magnet at the same polarity to induce a nonlinear magnetic repulsive force. A triboelectric harvester was integrated into the system by utilizing the lower surface of the tip magnet to serve as the top electrode of the harvester, while the bottom electrode with an attached polydimethylsiloxane insulator was placed underneath. Numerical simulations were performed to examine the impact of the potential wells formed by the magnets. The structure’s static and dynamic behaviors at varying excitation levels, separation distance, and surface charge density are all discussed. In order to develop a variable frequency system with a wide bandwidth, the system’s natural frequency varies by changing the distance between the two magnets to reduce or magnify the magnetic force to achieve monostable or bistable oscillations. When the system is excited by vibrations, the beams vibrate, which causes an impact between the triboelectric layers. An alternating electrical signal is generated from a periodic contact-separation motion between the harvester’s electrodes. Our theoretical findings were experimentally validated. The findings of this study have the potential to pave the way for the development of an effective energy harvester that is capable of scavenging energy from ambient vibrations across a broad range of excitation frequencies. The frequency bandwidth was found to increase by 120% at threshold distance compared to the conventional energy harvester. Nonlinear impact-driven triboelectric energy harvesters can effectively broaden the operational frequency bandwidth and enhance the harvested energy.

## 1. Introduction

Mechanical vibrations, such as those created by human motions, cars, and equipment, are significant sources of lost mechanical power in our daily environment [1]. Therefore, this is one of the most abundant forms of wasted energy. It could be converted into environmentally renewable energy and used to power widely used microelectronics in environment control, emergency response, the monitoring and control of industrial processes, health monitoring, implantable medical devices, ships, automobiles, drilling rigs, and many other applications [2,3,4,5,6,7]. The frequency bandwidth of environmental vibration energy is vast, with low-frequency components dominating [8]. Consequently, the thought of harnessing this ambient energy is enticing [9].

Recently, energy harvesting has been presented as a novel solution for converting wasted energy into usable electrical energy because of the advantages of being environmentally friendly with minimal maintenance costs compared to batteries as a power source. However, the currently proposed vibration energy harvesting systems still have a narrow operational bandwidth [10,11]. Multiple transduction systems have been employed to transform wasted mechanical energy into usable electricity, such as electromagnetic [12,13,14], piezoelectric [15,16,17,18], electrostatic [19,20,21], and triboelectric [22,23,24,25,26] energy. With low-cost materials, a high power density, environmental friendliness, an extended service life, and simple fabrication, triboelectric energy harvesting is considered an efficient method for converting small-scale kinetic energy into electricity [27]. In addition, the triboelectric energy harvesting technique has widespread applications utilizing many vibration sources, such as human activities [28,29,30], wind flow [31,32,33], and mechanical vibrations [23,34,35]. The working principle of triboelectricity is based on electrification and electrostatic induction, which happens when two materials with opposite polarities come into contact and then separate [36,37,38].

Even though external environmental excitations have a broad range, linear vibration energy harvesters have a narrow frequency bandwidth, which minimizes the amount of energy that can be harvested. Therefore, these harvesters must only be activated at resonance frequency to achieve a satisfactory energy conversion rate. Small deviations in the external excitation frequency from the resonance frequency of a linear vibration energy harvester substantially impact its performance, which makes them inefficient. In reality, by expanding the frequency bandwidth of a harvester, the system can be more effective across a broader spectrum of external frequencies [39,40]. Adding nonlinearity to the system is one of the most appropriate strategies for enhancing the frequency bandwidth of a harvester [41,42]. Several techniques for exploiting nonlinearity have been studied, including duffing [43,44,45,46], impact [47,48,49,50,51,52], and bistable oscillator designs [53,54,55,56,57,58]. Recently, vibro-impact has been applied to vibration energy harvesters to boost harvesting efficiency. Numerous technical applications use vibro-impact, including cutting and grinding equipment, pile-driving machines, turbomachinery, frequent rubbing of rotor blades and stators, and hand-held percussion devices [59]. Several studies investigated triboelectric and electrostatic effects utilizing vibro-impact structures and achieved higher bandwidth compared to the nonimpact harvesters [60]. The vibro-impact of multidegrees-of-freedom systems increased the operational bandwidth [51,61,62,63,64,65,66]. However, such systems need to be bulky and fabricated in large sizes. Other techniques were used to increase the output power and operating frequency bandwidth of piezoelectric and triboelectric energy harvester devices, such as mechanical impact [51,52,67] and mechanical stoppers [62,68,69,70]. However, all these previous studies have a specific range for operating frequencies, and changing the operating range requires modifying the structural parameters. Nonlinearities are another approach to extending the operating bandwidth of energy harvesters via bistability [35,55,58,71,72], compared to linear harvesters. However, the enhancement in the operating bandwidth utilizing only nonlinearity is still insignificant.

Nonlinearity plays a significant role in expanding the bandwidth of linear harvesters. Structural and magnetic nonlinearities are some of the most common techniques used to increase the frequency bandwidth and harvesting efficiency [73]. Scientists have devised numerous designs to produce bistable conditions, which broadens the response bandwidth and boosts the harvesting efficiency [74,75]. For example, a cantilever beam structure with two magnets generates monostable and bistable potential energy depending on the magnetic spacing fluctuation [55,58,76]. Nonlinear hysteresis (softening and hardening) induced large amplitude oscillations that significantly expanded the frequency bandwidth for both monostable [77] and bistable harvesters [78]. In addition, the inter-well motions due to the high kinetic energy in bistable energy harvester systems broadened the frequency bandwidth and enhanced the average power density [79]. Such bistable systems have been extensively explored and widely implemented in piezoelectric [80] and magnetoelectric energy harvesting [12], and electromagnetic energy harvesters [81]. A bistable actuator coupled with a flexible triboelectric nanogenerator sensor was designed to detect bladder fullness and help to empty it [82]. The work used permanent magnets and springs as a bistable structure in a TEH for broadband energy harvesting at low frequencies [83,84]. However, these studies in triboelectric did not shed light on the dynamic behavior of the harvesters, and in general, there is a lack of investigations into the static and dynamic behaviors of the mono, transition, and bistable regions in triboelectric energy harvesting systems.

We created a nonlinear variable frequency vibro-impact vibration energy harvester with triboelectric transducers to achieve a high energy density and large bandwidth under harmonic excitations. This study focused on combining magnetic nonlinearity with the inherent phenomenon of vibro-impact in triboelectric energy harvesters to broaden the harvesting bandwidth of triboelectric energy harvesters. The combination of magnetic nonlinearity and vibro-impact is a novel strategy for triboelectric energy harvester applications. Moreover, the addition of magnetic nonlinearity makes the harvester a variable frequency energy harvester, where the operating frequency can be controlled by controlling the distance between the two magnets to target multiple applications within the ambient range. In this study, we presented a variable frequency nonlinear vibro-impact energy harvester based on triboelectric with induced magnetic nonlinearity under harmonic excitations to increase the bandwidth. We contributed by combining magnetic nonlinearity with vibro-impact to enhance the operating bandwidth of linear vibro-impact harvesters. We developed a theoretical lumped piecewise model to comprehend the harvester’s static and dynamic behaviors. We experimentally validated our model to prove the viability of adding magnetic nonlinearity to the triboelectric transduction mechanism. This article is structured as follows: The design and configurations of triboelectric energy harvesters are covered in the Device Configuration Section and Principle of Operation. We constructed the theoretical model utilizing a lumped parameter model technique as outlined in the Theoretical Model Section, and the Results and Discussion Section provides the associated results, which were experimentally validated. In the Conclusion Section, we wrap up by drawing some conclusions.

## 2. Device Configuration and Principle of Operation

**Figure 1:**
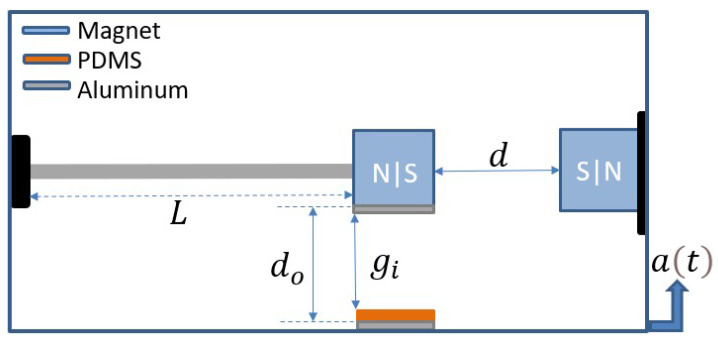
Schematic of the nonlinear energy harvester under base excitation.

A permanent magnet is attached to the tip of a cantilever beam. Another fixed magnet is positioned facing the tip magnet at the same polarity to induce magnetic nonlinearity to the structure. An aluminum layer is attached to the bottom surface of the tip magnet, which serves as an upper electrode of the triboelectric energy harvester. Another aluminum layer coated with polydimethylsiloxane (PDMS) is fixed below the upper electrode and acts as the lower electrode of the triboelectric generator. A schematic of the whole structure is shown in Figure 1. When the structure is subjected to base excitation, the cantilever beam vibrates, and the two electrodes contact each other periodically, generating an electrical signal. The two magnets are separated by a distance *d*, which determines the nature of the oscillations. At a large *d*, the magnetic force is weak, and the system oscillates around a single well, known as monostable oscillation. In contrast, at a low *d*, the magnetic force is magnified and the system oscillates in a double well, known as bistable oscillations. The separation distance that differentiates between the monostable from the bistable oscillation is known as the threshold distance ($d_{th}$).

**Figure 2:**
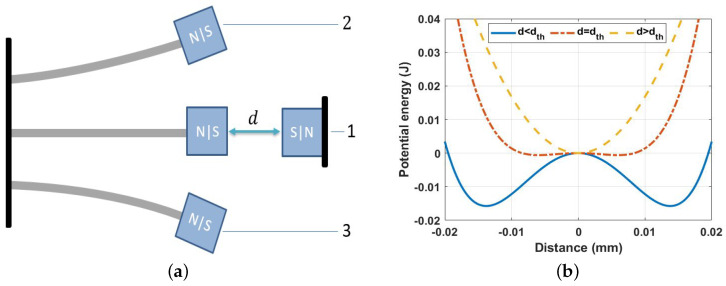
The principle of operation of the nonlinear harvester as a function of the distances between two magnets: (**a**) stability; (**b**) potential energy.

The principle of operation for the nonlinear harvester is shown in Figure 2. The stability of the harvester is a function of the distance between the two magnets, as shown in Figure 2a. The repulsive magnetic force becomes weak when the two magnets are set far from each other (large *d*), and the beam oscillates at a single stable equilibrium point around its horizontal axis (see case 1 in Figure 2a), which is called the monostable range. The corresponding potential energy function is shown in Figure 2b at a separation distance of $d>d_{th}$, where a single-potential well profile is shown and reflects the oscillation around case 1 in Figure 2a. In contrast, when the two magnets are set close to each other (small *d*), the repulsive magnetic force becomes strong and forces the beam to oscillate at double stable equilibrium points around its horizontal axis (cases 2 and 3 in Figure 2a), which is called the bistable range. The corresponding potential energy function is shown in Figure 2b at a separation distance of $d<d_{th}$, where double-potential well profiles are shown and reflect the oscillation around cases 2 and 3 in Figure 2a. From Figure 2b, we can see that the oscillations of the system are transferred from a single-potential well to a double-potential well by lowering the distance between the two magnets. Furthermore, the barrier between the double-potential wells becomes higher compared to the system’s energy, and then there are two degenerate states corresponding to the energy being localized in one well or the other.

**Figure 3:**
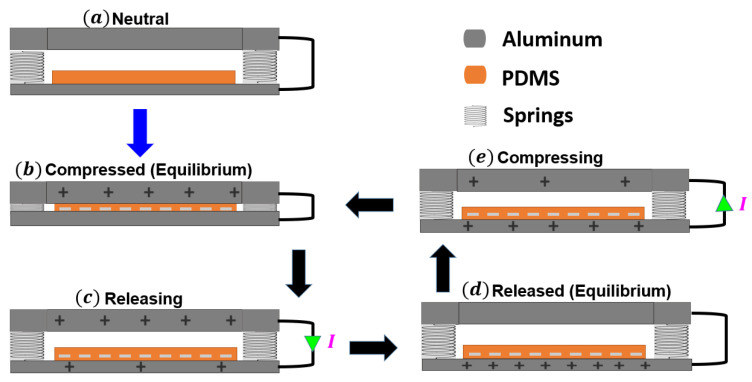
Triboelectric energy harvester cycle of work.

The triboelectric generator consists of two layers with opposing electron loss and gain tendencies and generates electricity through periodic contact and separation between the harvester’s electrodes. The triboelectric working mechanism generates electricity based on contact electrification and electrostatic induction. Therefore, the electrodes and the insulator are selected based on the triboelectric series, where the two materials should have an opposite tendency to lose and gain electrons. Our selection reflects this point since the aluminum tends to be positively charged, while the PDMS layer has the affinity to be negatively charged. The detailed working mechanism of the triboelectric generator is depicted in Figure 3. Initially, the harvester’s electrodes are neutral and free of charge; see Figure 3a. When the base excitation is strong enough to overcome the restoring force from the elastic beam, the harvester’s electrodes come into contact, the upper Al layer becomes positively charged, and the PDMS layer becomes negatively charged; see Figure 3b. Then, when the base excitation is weak, the restoring force from the elastic beam dominates, forcing the harvester’s electrodes to separate from each other, and the current flows from the upper Al layer to the lower one due to the potential difference between them; see Figure 3c. Once the electrostatic and triboelectric charges are liberated, they equalize and reach equilibrium; see Figure 3d. However, when the mechanical load is applied again, it breaks the equilibrium, capacitance is charged, and current flows in the opposite direction resulting in an alternating current being generated; see Figure 3e.

## 3. Theoretical Model

**Figure 4:**
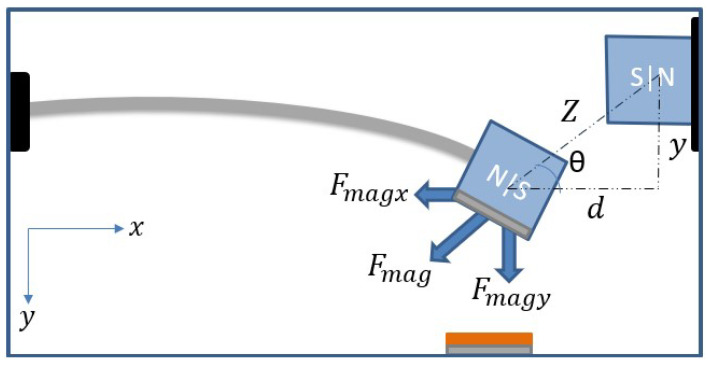
Schematic for the total magnetic force acting on tip magnets.

A lumped parameter model of an SDOF system was used to simulate the static and dynamic behavior and the generated electrical system. When the tip magnet faces another fixed magnet at the same polarity in the vibro-impact energy harvester, a repulsive magnetic force is induced between the two magnets. The total repulsive magnetic force ($F_{mag}$) is a function of the deflection of the beam, *y*, and the horizontal distance between the two magnets, *d*, and can be decomposed into horizontal and vertical components; see Figure 4. For simplicity, the horizontal component ($F_{magx}$) is neglected with the assumption that it is equivalent to the longitudinal stiffness of the cantilever beam. However, the vertical component ($F_{magy}$) is dominant and affects the transverse deflection of the cantilever beam.

The total magnetic force acting on both magnets is given by Equation (1), where *Z* is the distance between the two magnets’ centers and is given by ($Z=\sqrt{d^{2}+y^{2}}$). Further, $F_{R}$ is the size of the magnetic dipole moments and given by ( $F_{R}=\frac{3\epsilon q_{1}q_{2}}{2\pi }$), where $q_{1}$ and $q_{2}$ are the magnetic dipole moments for the two magnets. Using the angle θ, the vertical component ($F_{magy}$) is given by Equation (2), where μ is the permeability of the space and it equals $4\pi \times 10^{-7}$ m kg/s${}^{2}$ A${}^{2}$.

(1) $$F_{mag}=\frac{F_{R}}{Z^{4}}$$

(2) $$\begin{array}{c} F_{magy}=\frac{F_{R}y}{{\left(d^{2}+y^{2}\right)}^{5/2}} \end{array}$$

**Figure 5:**
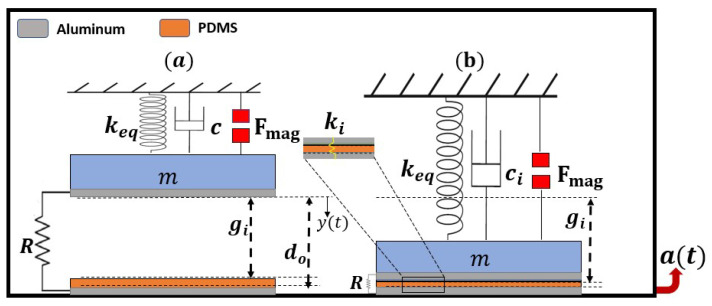
Single-degree-of-freedom vibration system (**a**) before impact and (**b**) at the start of the impact.

To analyze the static and dynamic characteristics of the nonlinear harvester, we employed a single-degree-of-freedom (SDOF) lumped parameter model, as depicted in Figure 5. The upper electrode of the triboelectric harvester is affixed to the bottom of the lumped mass and separated from the lower fixed electrode, which is attached to the PDMS layer, by an initial gap $g_{i}$. When subjected to base excitations, the system exhibits two motion scenarios: nonimpact and impact modes. The nonimpact mode takes place when the deflection of the upper electrode is smaller than the initial gap, as shown in Figure 5a. In contrast, if the deflection is equal to or greater than the initial gap, the upper Al electrode comes into contact with the PDMS layer and begins to penetrate it, indicating the impact mode, as demonstrated in Figure 5b. Subsequently, the system becomes stiffer and more damped, which is incorporated by introducing additional impact stiffness $k_{i}$ and impact damping $c_{i}$ to the system.

The theoretical governing equation can be extracted from the free body diagrams for the SDOF shown in Figure 5 with all forces acting on the lower electrode for the nonimpact and impact scenarios. Following the procedure presented in [52], the piecewise governing equations for the nonimpact and impact scenarios were extracted and are shown in Equation (8). The two electrodes of the triboelectric energy harvester serve as a parallel plate capacitor, and the third equation in Equation (8) represents the electrical domain equation responsible for electrical signal generation. The term *m* is the equivalent mass of the beam, and $k_{eq}$ is the equivalent stiffness of the cantilever beam with the mass of the tip, and is given by $k_{eq}=\frac{3EI}{L^{3}}$ [85]. The electrostatic force of the capacitor is given by $F_{e}=\frac{q^{2}\left(t\right)}{2\epsilon_{0}\epsilon_{r}S}$, where q(t) is the number of charges carried between two electrodes and can be expressed as [52], *S* is the entire surface area of contact, $\epsilon_{r}$ is the dielectric constant of the PDMS, and $\epsilon_{0}$ is the vacuum permittivity. The term a(t) represents the harmonic base excitation and is equal to a(t)=Acos(Ωt), where *A* is the amplitude, and Ω is the excitation frequency. Moreover, $c_{i}$ and $k_{i}$ indicate the coefficients of impact damping and stiffening, respectively. In addition, $g_{i}$ is the distance between the upper electrode and the PDMS surface, wherein $d_{0}$ represents the distance between the two Al electrodes. The terms σ, *T*, and *R* represent the surface charge density, PDMS thickness, and external resistance, respectively. The rest of the parameters used in this study are listed in Table 1. Next, the governing equations can be solved numerically to examine the dynamic behavior of the system and the generated electrical signal.

(3) $$\begin{array}{c} \left\{\begin{array}{cc} m\ddot{y}+c\dot{y}+k_{eq}y-F_{magy}+F_{e}=ma, & y\left(t\right)<g_{i}\\ m\ddot{y}+c_{i}\dot{y}+k_{eq}y+k_{i}\left(y\left(t\right)-g_{i}\right)-F_{magy}=ma, & y\left(t\right)\geq g_{i} \end{array}\right.\\ \dot{q}=-\frac{q(t)}{\epsilon_{0}RS}\left(\frac{T}{\epsilon_{r}}+d_{0}-y\left(t\right)\right)+\frac{\sigma }{\epsilon_{0}RS}\left(d_{0}-y\left(t\right)\right) \end{array}$$

## 4. Experimental Setup

**Figure 6:**
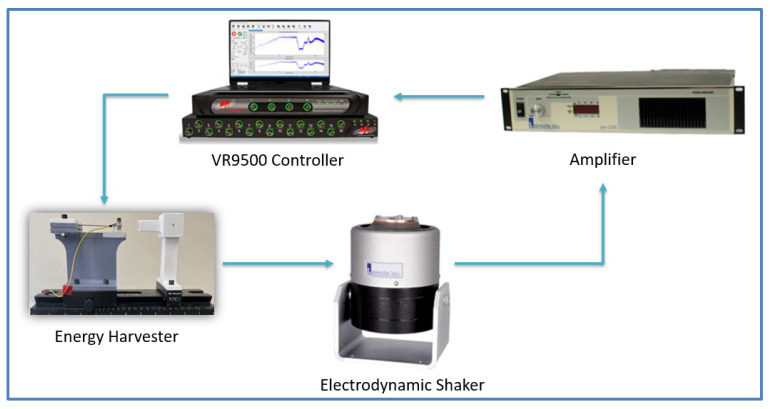
The experimental setup for testing the triboelectric energy harvester.

To validate our theoretical model, the experimental setup shown in Figure 6 was used to test the static and dynamic behaviors of the nonlinear harvester. The arrangement consisted of a VR9500 controller, an amplifier, and an electrodynamic shaker with a mounted nonlinear energy harvester structure. The control unit regulated the base excitation applied by the shaker to control the amplitude and frequency. The control unit sent the signal to the amplifier for amplification at the required level to be sent to the shaker, which transferred the base excitations to the harvester. An accelerometer was attached to the cantilever beam’s tip mass to record deflections in response to sweeping excitation frequencies. In addition, the voltage generated by the triboelectric generator was measured by the controller.

## 5. Results and Discussion

In this section, the system is numerically solved and experimentally validated to facilitate further analysis, to identify the crucial elements that lead to a more effective energy harvester, and to examine the potential for energy scavenging in the proposed harvester.

### 5.1. Static Analysis

**Figure 7:**
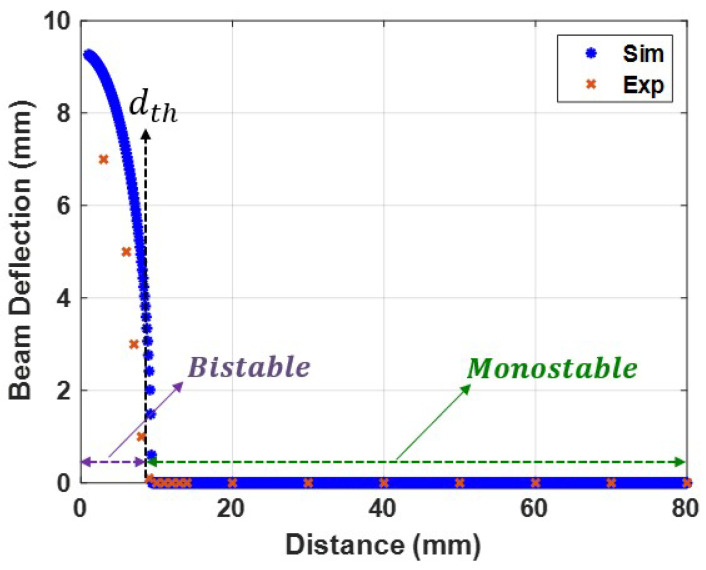
Experimental and theoretical static response of the beam as a function of the separation between the two magnets (*d*). The threshold distance ($d_{th}$) was found to be 9 mm.

The static deflection of the beam is a function of the distance between the two magnets due to the repulsive magnetic force. By setting all the time derivatives to zero in Equation (8), the static governing equation can be given by:

(4) $$ky_{s}-F_{magys}=0$$

where

(5) $$F_{magys}=\frac{F_{R}y_{s}}{{\left(d^{2}+y_{s}^{2}\right)}^{5/2}}$$

where $y_{s}$ represents the static deflection of the cantilever beam, and $F_{magys}$ is the static magnetic force extracted from Equation (2). The static solution for Equation (4) is calculated based on the geometric parameters given in Table 1. The static deflection of the cantilever beam varies with the separation of two magnets, as seen in Figure 7. It is demonstrated conclusively that the static response had a critical threshold separation distance $d_{th}$ of 9 mm, dividing the static profile into monostable ($d>d_{th}$) and bistable ($d\leq d_{th}$) regions. The monostable regime had a single stable branch for the static response, whereas the bistable regime had two stable branches (upper and lower). Due to the symmetry and the restriction from the lower electrode, only one branch of the horizontal beam’s stable solution is presented in Figure 7, where the maximum static deflection of the cantilever beam found to be at the bistable regime was 9.3 mm. Furthermore, the static deflections as a function of the separation distance between the two magnets were measured experimentally, and the results in Figure 7 show a good agreement with the theoretical results.

### 5.2. Dynamic Analysis

#### 5.2.1. Natural Frequencies

Next, we examine the effect of the magnetic force on the harvester’s natural frequency. Herein, the total deflection of the beam is taken to be a function of the static and dynamic deflections as ($y=y_{s}+y_{u}$), where $y_{u}$ is the dynamic deflection of the beam. By substituting this in Equation (3), we arrive at the following system of equations:

(6) $$\begin{array}{c} \left\{\begin{array}{cc} m\ddot{y_{u}}+c\dot{y_{u}}+k_{eq}y_{u}+k_{eq}y_{s}-F_{magyus}+F_{e}=ma, & y_{u}\left(t\right)<g_{i}\\ m\ddot{y_{u}}+c_{i}\dot{y_{u}}+k_{eq}y_{u}+k_{eq}y_{s}+k_{i}\left(y_{u}\left(t\right)+y_{s}-g_{i}\right)-F_{magyus}=ma, & y_{u}\left(t\right)\geq g_{i} \end{array}\right.\\ \dot{q}=-\frac{q(t)}{\epsilon_{0}RS}\left(\frac{T}{\epsilon_{r}}+d_{0}-y_{u}\left(t\right)-y_{s}\right)+\frac{\sigma }{\epsilon_{0}RS}\left(d_{0}-y_{u}\left(t\right)-y_{s}\right) \end{array}$$

where $F_{magyus}$ is given by:

(7) $$\begin{array}{c} F_{magyus}=\frac{F_{R}\left(y_{u}+y_{s}\right)}{{\left(d^{2}+{\left(y_{u}+y_{s}\right)}^{2}\right)}^{5/2}} \end{array}$$

(8) $$\left\{\begin{array}{cc} m\ddot{y}+c\dot{y}+k_{eq}y-F_{magy}+F_{e}=ma\left(t\right), & y\left(t\right)<g_{i}\\ m\ddot{y}+c_{i}\dot{y}+k_{eq}y+k_{i}\left(y\left(t\right)-g_{i}\right)-F_{magy}=ma\left(t\right), & y\left(t\right)\geq g_{i}\\ \dot{q}=-\frac{q(t)}{\epsilon_{0}RS}\left(\frac{T}{\epsilon_{r}}+d_{0}-y\left(t\right)\right)+\frac{\sigma }{\epsilon_{0}RS}\left(d_{0}-y\left(t\right)\right) \end{array}\right.$$

To eliminate the static effect and avoid the system’s complexity while obtaining the numerical solution, the magnetic force, $F_{magyus}$, is expanded with Taylor’s series around zero dynamic deflection $y_{u}=0$ as follows:

(9) $$\begin{array}{cc} F_{magyus} & =\frac{F_{R}y_{s}}{{\left(d^{2}+y_{s}^{2}\right)}^{5/2}}+\alpha_{1}y_{u}+\alpha_{2}y_{u}^{2}+\alpha_{3}y_{u}^{3}+\ldots \\ \; & =F_{magys}+\alpha_{1}y_{u}+F_{magyu} \end{array}$$

where $F_{magyu}$ is the dynamic portion of the expanded magnetic force omitting the linear term, and $\alpha_{i}$ represents the coefficient components of the magnetic force after Taylor series expansion. The first nine terms are listed in Appendix A (Equation A1). The final form of the dynamic governing equation is obtained by substituting Equation (9) into Equation (6) and then canceling the static terms using Equation (4). This results in the following:

(10) $$\begin{array}{c} \left\{\begin{array}{ll} m{\ddot{y}}_{u}+c{\dot{y}}_{u}+\left(k_{eq}-\alpha_{1}\right)y_{u}-F_{magyu}+F_{e}=ma, & y_{u}\left(t\right)<g_{i}\\ m{\ddot{y}}_{u}+c_{i}{\dot{y}}_{u}+\left(k_{eq}-\alpha_{1}\right)y_{u}+k_{i}\left(y_{u}\left(t\right)+y_{s}-g_{i}\right)-F_{magyu}=ma, & y_{u}\left(t\right)\geq g_{i} \end{array}\right.\\ \dot{q}=-\frac{q(t)}{\epsilon_{0}RS}\left(\frac{T}{\epsilon_{r}}+d_{0}-y_{u}\left(t\right)-y_{s}\right)+\frac{\sigma }{\epsilon_{0}RS}\left(d_{0}-y_{u}\left(t\right)-y_{s}\right) \end{array}$$

According to Equation (10), the natural frequency of the nonlinear resonator under the effect of the magnetic force is calculated as:

(11) $$\begin{array}{c} f_{n}=\frac{1}{2\pi }\sqrt{\frac{k_{eq}-\alpha_{1}}{m}} \end{array}$$

where $\alpha_{1}$ is the linear coefficient of the dynamic magnetic force after the Taylor series expansion of Equation (7) around y=0, and given by:

(12) $$\begin{array}{c} \alpha_{1}=\frac{F_{R}\left(d^{2}-4y_{s}^{2}\right)}{{\left(d^{2}+y_{s}^{2}\right)}^{7/2}} \end{array}$$

**Figure 8:**
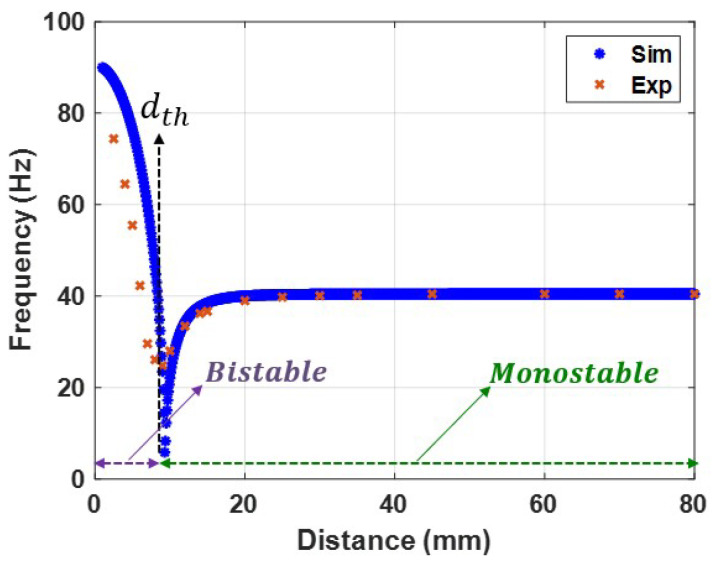
Variation in natural frequency with distance *d* between two magnets at 0.1 g.

**Figure 9:**
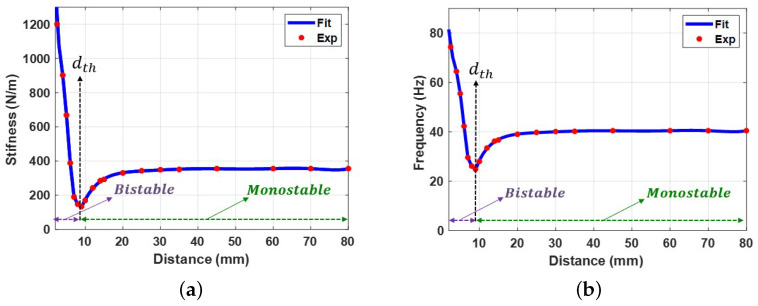
(**a**) Variation in stiffness with distance *d* between two magnets. (**b**) Variation in natural frequency with distance *d* between two magnets.

It is possible to determine the relationship between the natural frequency and the magnetic force by varying the distance between the two magnets and calculating the natural frequency value using Equation (11). The natural frequency variation with the separation distance between the two magnets is shown in Figure 8. The results show that the bistable and monostable zones were separated by a threshold distance of 9 mm, which is consistent with the static results presented in Figure 7. The system was in the bistable region at low separation distances, where the magnetic force exerted the most substantial effect. In this zone, the natural frequency reached a higher frequency than the linear natural frequency and reached a maximum value of 90 Hz. The natural frequency was gradually decreased by increasing the distance between the two magnets until it reached its minimum value at the threshold distance. Moreover, the system was tested experimentally at a 0.1 g excitation level, and the experimental variance of the natural frequencies with separation distance was obtained and is presented in Figure 8. The experimental and simulated findings are in good agreement in the monostable range but not in perfect agreement in the transition and bistable ranges. The reason behind this difference could be the low accuracy of the SDOF model at high nonlinearities. Another reason could be the experimental measurement errors. This slight difference between the experimental and simulated values of the natural frequencies would cause significant mismatch problems later when investigating the dynamic behavior of the harvester when we tried to validate our theoretical results. Therefore, this difference needed to be eliminated. To this end, the experimental results for the variation of the natural frequencies were used to extract the experimental stiffness values shown in Figure 9a. Then, a piecewise curve fit function for the stiffness as a function of the separation distance between the two magnets was extracted and is shown in Equation (13). The piecewise stiffness function was then used to calculate the natural frequencies, which greatly agree with experimental results, as shown in Figure 9b. Furthermore, the piecewise stiffness function is used in the further analysis of the dynamic behavior of the harvester.

(13) $$k=\left\{\begin{array}{cc} 5956.75-4954.35d+2004.2d^{2}-398d^{3}+37.16d^{4}-1.31d^{5} & \;\mathrm{if}\;d<d_{th}\\ -937.71+224.567d-16.7d^{2}+0.67d^{3}-0.01565d^{4}+0.000212d^{5}-1.5343\times 10^{-6}d^{6}+4.61\times 10^{-9}d^{7} & \;\mathrm{if}\;d\geq d_{th} \end{array}\right.$$

#### 5.2.2. Linear and Conventional Harvester Analysis

**Figure 10:**
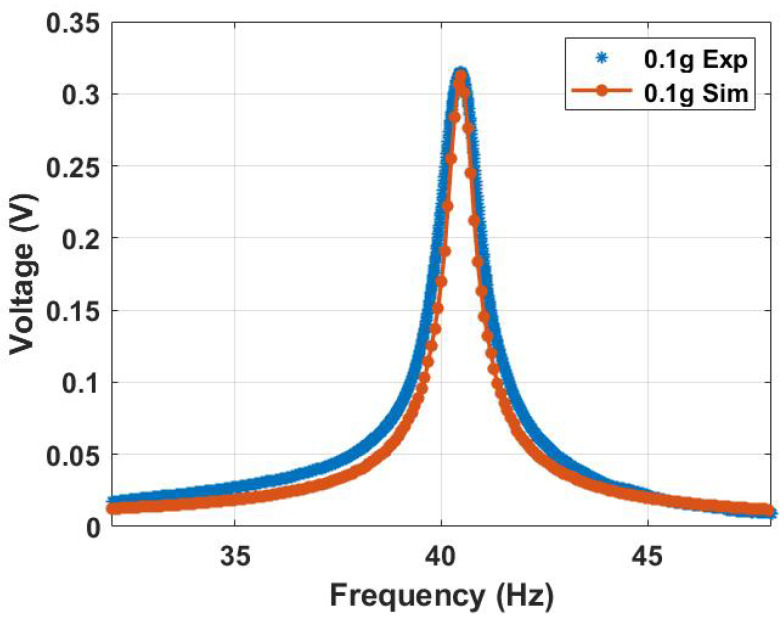
The linear experimental and theoretical frequency voltage curve without the influence of the magnetic force at a low excitation level of 0.1 g, c=0.03, and σ = 1.3 μC/m${}^{2}$.

Eliminating the influence of magnetic nonlinearity by setting $F_{magyu}$ equal to zero allowed for the investigation of the linear harvester behavior and the conventional vibro-impact harvester. First, the linear harvester was investigated by exciting the system at a low excitation level of 0.1 g, which was insufficient to introduce impact between the harvester’s electrodes. The linear frequency voltage curve is shown in Figure 10. The theoretical and experimental results are in good agreement. They show that the natural frequency of the harvester had a natural frequency of approximately 40.4 Hz.

**Figure 11:**
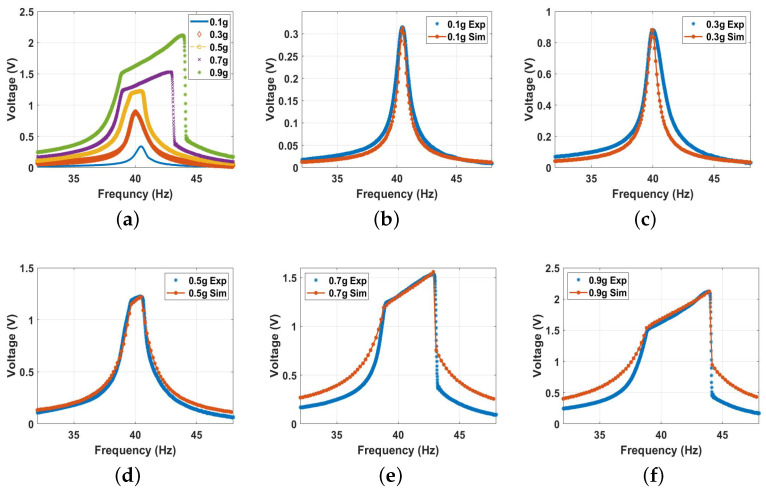
The frequency voltage curve of the beam at different excitation levels without magnetic force effect: (**a**) experimental results; (**b**) 0.1 g, c=0.03, and σ = 1.35 μC/m${}^{2}$; (**c**) 0.3 g, c=0.0335, and σ = 1.4 μC/m${}^{2}$; (**d**) 0.5 g, c=0.05, and σ = 2.8 μC/m${}^{2}$; (**e**) 0.7 g, c=0.03, and σ = 4.2 μC/m${}^{2}$; (**f**) 0.9 g, c=0.03, and σ = 5.2 μC/m${}^{2}$.

Second, the system was excited with higher excitation levels to investigate the behavior of the conventional vibro-impact harvester. The experimental voltage frequency curves at different excitation levels are shown in Figure 11a. The results show an increment in the output voltage with increasing the excitation level, reaching a maximum value of 2.2 V at an excitation level of 0.9 g. Furthermore, the results in Figure 11a depict a significant increase in the bandwidth due to the impact between the harvester’s electrodes starting at the 0.5 g excitation level and increasing significantly at higher excitation levels. In addition, the results in Figure 11a show a softening behavior at low excitation levels (≤0.3 g), where the system’s natural frequency was shifting to lower values to the left, while at higher excitation levels (≥0.3 g), the frequency tended to be shifted to a higher value to the right, indicating hardening behavior. The combination of both behaviors is known as combined behavior [55]. The experimental results and how they match with the simulated results for each excitation level separately are shown in Figure 11b,c, where the theoretical and experimental findings are in good agreement for all cases.

#### 5.2.3. Nonlinear Analysis

**Figure 12:**
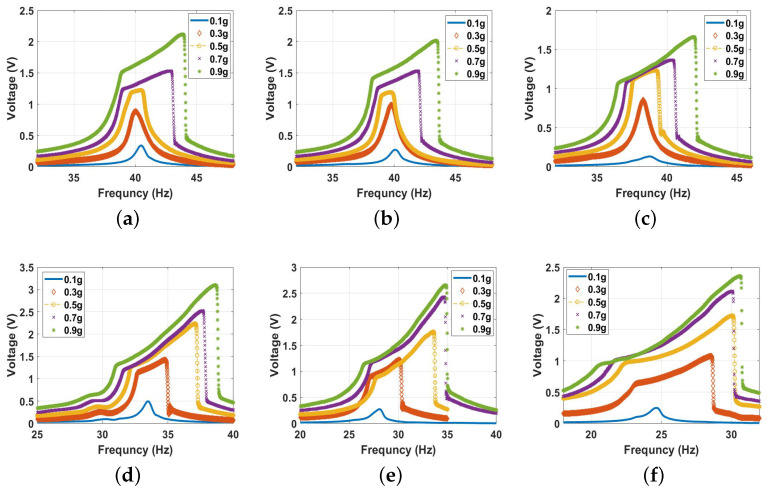
The frequency voltage curve of the beam at different excitation levels and for selected magnet spacings of: (**a**) No magnet; (**b**) d=30 mm; (**c**) d=20 mm; (**d**) d=12 mm; (**e**) d=10 mm; (**f**) d=9 mm.

**Figure 13:**
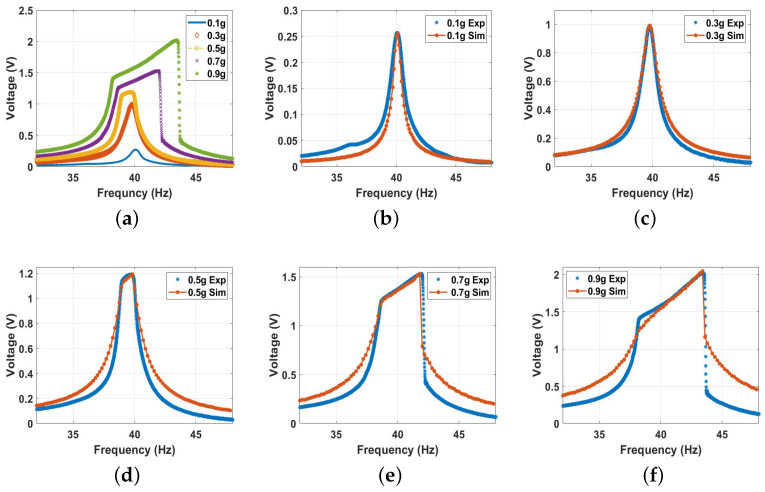
The voltage frequency response curve of the monostable range at d=30mm at different excitation levels: (**a**) experimental results; (**b**) 0.1g, c=0.02, and σ = 1.06 μC/m${}^{2}$; (**c**) 0.3g, c=0.04, and σ = 2.7 μC/m${}^{2}$; (**d**) 0.5g, c=0.05, and σ = 3.9 μC/m${}^{2}$; (**e**) 0.7 g, c=0.009, and σ = 2.96 μC/m${}^{2}$; (**f**) 0.9 g, c=0.001, and σ = 2.9 μC/m${}^{2}$.

In this section, we investigate the dynamics of the energy harvester under the influence of the magnetic force in the monostable, bistable, and transition zones. The distance between the two magnets was selected to achieve the required range, while the voltage responses was analyzed at various excitation levels. The experimental results at different magnetic spacing are shown in Figure 12. First, we investigated the dynamics of the energy harvester in the monostable zone starting with a separation distance of 30 mm. The experimental voltage frequency curves with the corresponding simulated results for different excitation levels are shown in Figure 13 and show good agreement. Comparing the results at 30 mm (Figure 13) with the previous results under no magnetic effect (Figure 11), we notice that the results are quite close to each other. This is because the two magnets were far from each other at this distance, and the magnetic force was feeble, so its effect was almost neglected. It should also be noted that greater excitation levels resulted in a higher output voltage and bandwidth. Moreover, the combined behavior is shown in the results with softening behavior being dominant at low excitation levels. In contrast, the hardening behavior was dominant at higher excitation levels.

**Figure 14:**
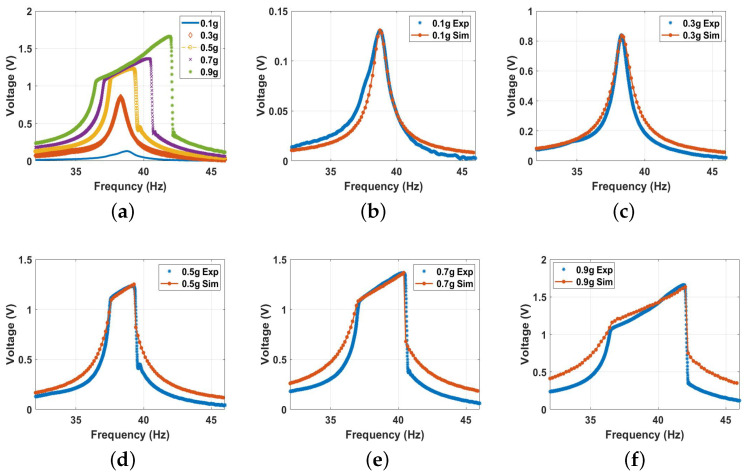
The voltage frequency response curve of the monostable range at d=20 mm at different excitation levels: (**a**) experimental results; (**b**) 0.1 g, c=0.035, and σ = 0.91 μC/m${}^{2}$; (**c**) 0.3 g, c=0.04, and σ = 2.2 μC/m${}^{2}$; (**d**) 0.5 g, c=0.035, and σ = 2.68 μC/m${}^{2}$; (**e**) 0.7 g, c=0.02, and σ = 2.93 μC/m${}^{2}$; (**f**) 0.9 g, c=0.01, and σ = 3.5 μC/m${}^{2}$.

Next, we set the distance between the two magnets to 20 mm to investigate the dynamic behavior in the monostable range, but at a stronger magnetic force influence. The results in Figure 14 show the frequency voltage curves extracted experimentally and matched with the simulations from the theoretical model. They are in good agreement. According to the results shown in Figure 14, several things can be observed. First, the natural frequency was shifted to the left to reach a lower value of 38.7 Hz compared to the conventional and 30 mm frequencies. This shift was due to the effect of the higher magnetic nonlinearity at this distance compared to the previous cases. This led to the beam having a lower natural frequency according to the results presented in Figure 9b. Second, larger bandwidth was achieved by increasing the excitation level, where the impact between the harvester became more significant, with a wider range of frequencies compared to the low excitation levels. In addition, in the 20 mm case, the bandwidth was slightly larger than the conventional and 30 mm bandwidth. This slight difference was because, even though the magnetic force was stronger than those in the previous cases, it was still considered feeble. Third, the output voltage increased with the excitation level, which can also be related to the higher impact between the harvester electrodes at higher excitations. However, the output voltage at this distance was less than the conventional and 30 mm output voltage.

**Figure 15:**
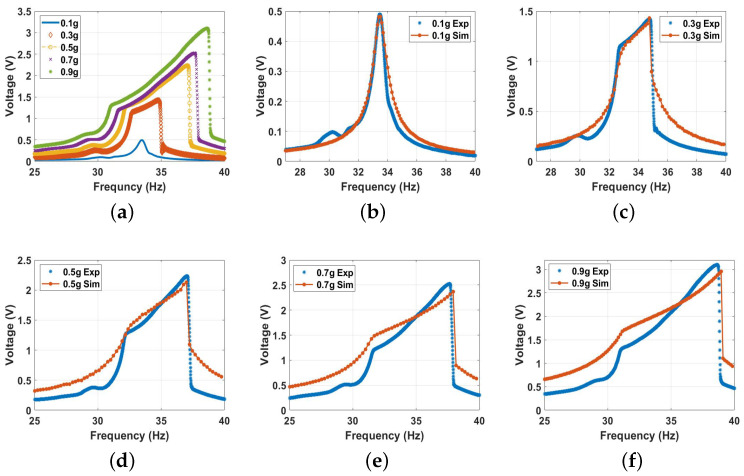
The voltage frequency response curve of the monostable range at d=12 mm at different excitation levels: (**a**) experimental results; (**b**) 0.1 g, c=0.03, and σ=2.5 μC/m${}^{2}$; (**c**) 0.3 g, c=0.001, and σ=2.6 μC/m${}^{2}$; (**d**) 0.5 g, c=0.003, and σ=4.7 μC/m${}^{2}$; (**e**) 0.7 g c=0.02, and σ=6.4 μC/m${}^{2}$; (**f**) 0.9 g, c=0.04, and σ=7.4 μC/m${}^{2}$.

By lowering the separation distance between the two magnets more, particularly to 12 mm, the system entered the transition range from the monostable side. The frequency voltage curves for a 12 mm distance at different excitations from both the experimental and simulations are shown in Figure 15 with a good agreement. The results show a shift in the natural frequency to a lower value of 33.5 Hz, indicating softening behavior. Moreover, a higher bandwidth was achieved even at lower excitations, where the impact between the harvester’s layers started at 0.3 g compared to 0.5 g in all the previous cases and increased significantly at higher excitation levels. A maximum bandwidth of 7.5 Hz was achieved at 0.9 g compared to 5.0 Hz in the previous cases at the same excitation level, which was equivalent to a 50% increment. This increment in the bandwidth at a low excitation was due to the effect of the magnetic force, which started to be stronger and more significant at a lower distance. In addition, the output voltage increased significantly by 44.3% with the increasing excitation level to reach a maximum value of 3.2 V compared to a maximum output voltage of 2.2 V from the conventional harvester at the same excitation level. Furthermore, hardening behavior started to appear by increasing the excitation level, which indicates the dominance of the positive cubic nonlinearity from the magnetic force, shifting the whole behavior to the right.

**Figure 16:**
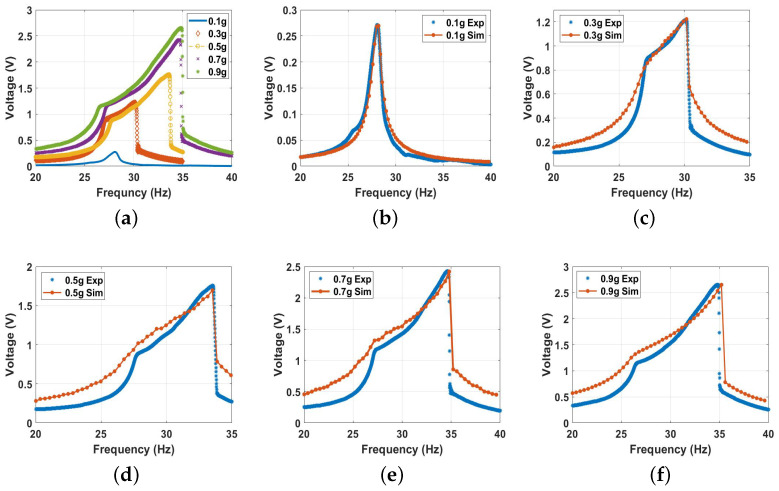
The voltage frequency response curve of the monostable range at d=10 mm at different excitation levels: (**a**) experimental results; (**b**) 0.1 g, c=0.03, and σ=1.19 μC/m${}^{2}$; (**c**) 0.3 g, c=0.001, and σ=2.45 μC/m${}^{2}$; (**d**) 0.5 g, c=0.0035, and σ=4.1 μC/m${}^{2}$; (**e**) 0.7 g, c=0.006, and σ=5.2 μC/m${}^{2}$; (**f**) 0.9 g, c=0.03, and σ=5.65 μC/m${}^{2}$.

Lowering the separation distance to 10.0 mm resulted in lowering the natural frequency to a lower value, and the softening behavior became more significant. In addition, a wider bandwidth was achieved compared to the previous cases, as shown in Figure 16. However, the voltage amplitude exhibited a reduction compared to the 12 mm case, which could be due to the effect of the static magnetic force reducing the gap between the two electrodes, resulting in a broader bandwidth, and, at the same time, restricting the beam from oscillating freely.

**Figure 17:**
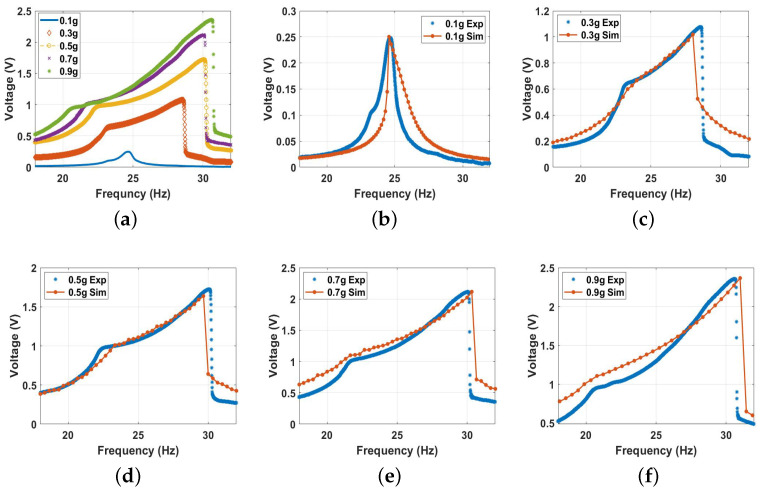
The voltage frequency response curve of the threshold at d=9 mm at different excitation levels: (**a**) experimental results; (**b**) 0.1 g, c=0.03, and σ=1.01 μC/m${}^{2}$; (**c**) 0.3 g, c=0.001, and σ=2.4 μC/m${}^{2}$; (**d**) 0.5 g, c=0.0065, and σ=4 μC/m${}^{2}$; (**e**) 0.7 g, c=0.008, and σ=4.4 μC/m${}^{2}$; (**f**) 0.9 g, c=0.025, and σ=4.6 μC/m${}^{2}$.

By further decreasing the distance to reach the threshold at $d_{th}$, the bandwidth increased even more than all the previous cases at the same excitation levels, as shown in Figure 17. However, this increment in the bandwidth was at the cost of the voltage output since it dropped to a maximum value of 2.4 V at 0.9 g. The drop in the voltage out was expected because the natural frequency reached the lowest value of 24.8 Hz, which matches the results in Figure 9b. This drop in the natural frequency to a lower value indicates softening behavior compared to the linear harvester. It was due to the predominance of quadratic nonlinearity at the threshold distance.

**Figure 18:**
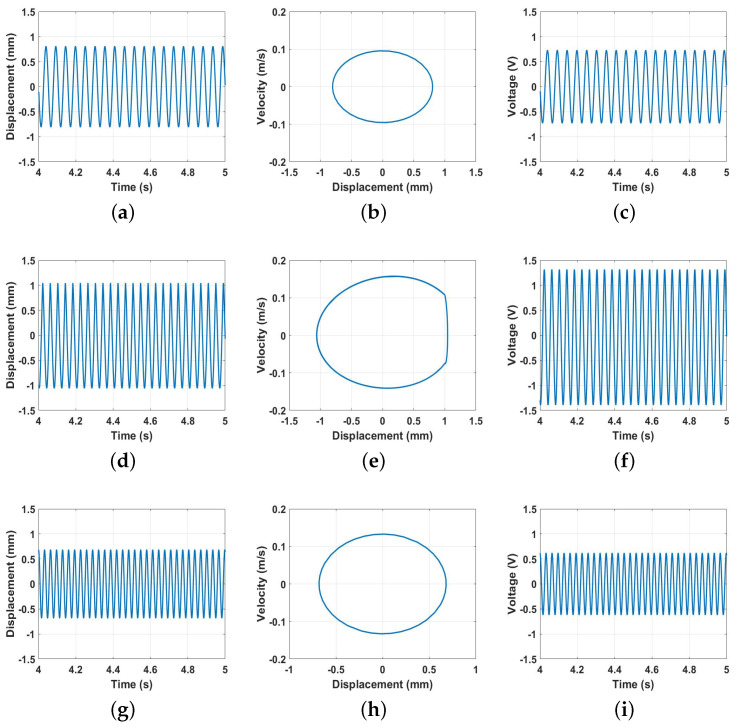
Response and the output voltage at a variety of frequencies when the distance *d* was set at 9 mm. (**a**) Time-response at 19 Hz, (**b**) Phase portrait at 19 Hz, (**c**) Time-voltage at 19 Hz, (**d**) Time-response at 24.8 Hz, (**e**) Phase portrait at 24.8 Hz, (**f**) Time-voltage at 24.8 Hz, (**g**) Time-response at 31.06 Hz, (**h**) Phase portrait at 31.06 Hz, (**i**) Time-voltage at 31.06 Hz.

In order to further investigate the dynamic behavior of the harvesting system at the threshold distance, the harvester’s time response, phase portrait, and time–voltage/velocity were extracted at specific frequencies to reflect the response in the before-impact, during-impact, and after-impact zones, as shown in Figure 18. Before the impact, at 19 Hz, the maximum harvester oscillation was around 0.8 mm, which is less than the gap between the harvester’s electrodes (1.0 mm), as shown in Figure 18a. The corresponding phase portrait and voltage output are shown in Figure 18b,c, respectively. The phase portrait shows a stable oscillation without any sudden change in the velocity, which is an indication of no impact at this frequency. The voltage output in Figure 18c is small since it was only due to the effect of the capacitance. By increasing the excitation frequency to 24.8 Hz, the oscillations reached the gap distance (Figure 18d) and the during-impact zone occurred between the harvester electrodes, as shown by the sudden change in the velocity and the restriction of the displacement in the phase portrait; see Figure 18e. This impact activates the contact electrification process, and higher voltage output can be generated, as shown in Figure 18f. When the excitation frequency exceeded 31.06 Hz, the system entered the after-impact zone, which is similar in behavior to the before-impact zone, as shown in Figure 18g−i.

**Figure 19:**
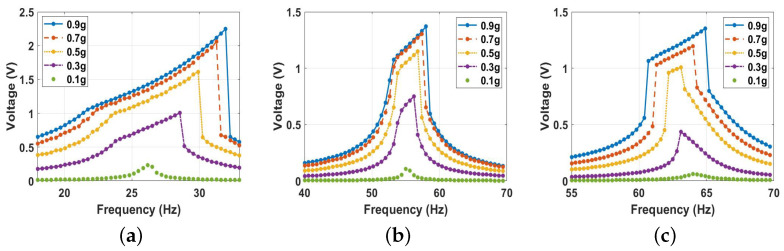
The voltage frequency response curve of the bistable at: (**a**) d=8 mm; (**b**) d=5 mm; (**c**) d=4 mm.

At a distance below the threshold value, the magnetic nonlinearity became very strong. Experimentally and under the base excitations, it deflected the beam to the degree where the repulsive magnetic force between the two magnets became significantly large and caused the tip magnet to stick to the fixed one, leading to a failure in the system. Therefore, since we validated our theoretical model experimentally, the dynamic behavior of the energy harvester in the bistable range was explored through the theoretical model only. Specifically, the distance between the two magnets was lowered to 8 mm, 5 mm, and 4 mm, as shown in Figure 19. At 8 mm (Figure 19a), the system still showed an impact behavior close to the range of frequencies at the threshold. In addition, the generated voltage dropped significantly to 2.24 V. Furthermore, a hardening behavior is shown by increasing the excitation level. Lowering the separation distance to the lower values of 5 mm and 4 mm increased the natural frequencies to reach higher values of 55.3 Hz and 64.25 Hz, respectively, indicating a hardening behavior. This significant increment in the natural frequency in the system was due to the strong magnetic nonlinearity induced at lower separation distances. However, increasing the excitation level forced the system to exhibit softening behaviors, where the quadratic nonlinearity became dominant, as shown in Figure 19b,c. Furthermore, the impact bandwidth became smaller at 5 mm and 4 mm, while the output voltage reached the lowest values compared to all the previous cases. Even though the system reached higher frequencies at lower distances, the output voltage was minimized, possibly due to the high magnetic force, which forced the beam’s tip magnet to become stuck to one of the potential wells and restrict its oscillations. Therefore, it was hypothesized that the deflections would be smaller than the distance between the triboelectric harvester’s electrodes.

**Figure 20:**
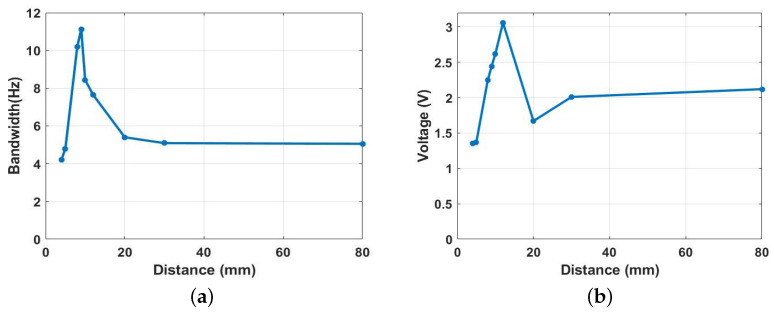
(**a**) Expanded bandwidth vs. magnet separation distance at 0.9 g. (**b**) Voltage output vs. magnet separation distance at 0.9 g.

Next, to have an indication of the optimal range for the proposed harvester, we calculated the impact bandwidth and voltage output of the harvester at a selected excitation level of 0.9 g for all the previous cases of the separation distance between the two magnets, and the results are summarized in Figure 20. The bandwidth was maximized in a range before and after the threshold distance (9.0 mm): this range is known as the transition regime. It was found that, at an excitation level of 0.9 g, an increment of around 120% in the bandwidth was achieved at the transition region of the nonlinear harvester compared to the conventional harvester presented in Figure 11f. Furthermore, the output voltage was maximized at the transition region, as shown in Figure 20b. Therefore, the transition region is considered the optimal region for the harvester to operate since the bandwidth and voltage output are maximized, allowing more energy to be harvested from the ambient excitations.

#### 5.2.4. Parametric Analysis

From a design point of view, the harvester should be designed to achieve higher amplitudes and maintain a wider bandwidth; thus, finding a compromise to accommodate both needs to be taken into consideration. However, since the goal is to expand the bandwidth and achieve the highest outputs, in this section, we parametrically investigate the influence of some critical parameters that could be used to enhance the design to achieve higher amplitudes at a wider operating bandwidth.

**Figure 21:**
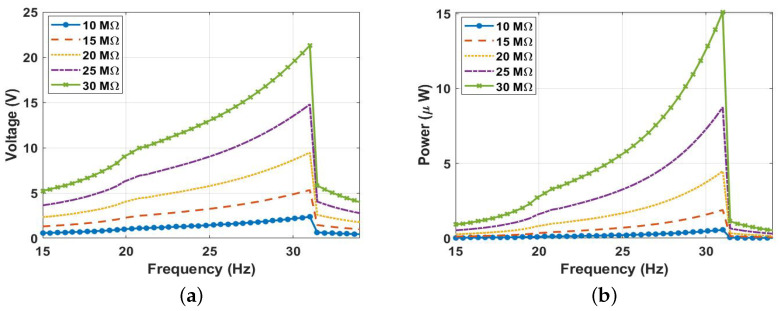
(**a**) The frequency-voltage, and (**b**) the frequency-power curves at 0.9 g excitation level and 9 mm separation distance at various resistances (*R*).

It is essential to explore the effect that external resistance has on harvester performance since it directly influences the amount of power produced. To do this, we fixed the distance between the two magnets at d=9 mm and varied the resistance from 10 MΩ to 30 MΩ at a 0.9 g excitation level, as seen in Figure 21a. As a result, a higher voltage signal was generated at a higher external resistance, with a maximum of 21.5 V at a resistance of 30 MΩ. Moreover, the output power was calculated at different resistance values, as shown in Figure 21b. As a result, it was demonstrated that the power increased by increasing the resistance until it reached its maximum value of 15 μW at a resistance of 30 MΩ. However, a further increment in the external resistance continued to increase the output power until it reached the maximum possible power, where the external resistance matched the internal resistance of the harvester. Beyond this value, the output power started to drop. Therefore, it is worth mentioning that the internal resistance needs to be extracted for practical applications to enhance the triboelectric energy harvester’s performance and maximize the power output.

**Figure 22:**
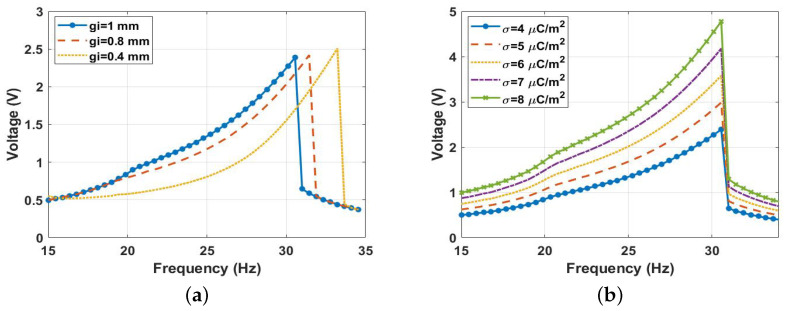
The frequency voltage curve at 9 mm of separation distance: (**a**) redvarious gap distances $g_{i}$; (**b**) various surface charge densities (σ).

To further investigate the effect of the parameters on harvester performance, we investigated the impact of the gap distance between the harvester’s electrodes. To do this, the system was excited at a 0.9 g excitation level for various values of the gap distance, as shown in Figure 22a. This shows that a wider bandwidth was possible at lower gaps, while greater amplitudes could be achieved at higher gaps but at the expense of bandwidths. At lower gaps, more contact occurred at a wider range of frequencies, resulting in a wider bandwidth. Conversely, higher gaps led to less contact at a narrower frequency range resulting in a limited bandwidth. Moreover, the surface charge density was an additional important component that substantially impacted the magnitude of the voltage generated. The voltage amplitude fluctuation under various surface charge density values is depicted in Figure 22b at an excitation level of 0.9 g, and magnets spacing of 9 mm, while all other parameters were held constant. The results indicate that, as the charge density increased, the voltage amplitudes grew significantly. It is important to note that the surface charge density is a function of the surface area of contact between the triboelectric layers. Designing such layers with microsurface patterns is one of the primary characteristics that could increase this charge density and result in a higher generated voltage.

## 6. Conclusions

This study describes a method for dramatically improving the performance of bistable triboelectric energy harvesters by combining magnetic nonlinearity and vibro-impact. The system comprises a cantilever beam with a tip magnet facing another fixed magnet at the same polarity to create a repulsive magnetic force. In addition, a triboelectric energy harvester was embedded in the system to create a vibro-impact triboelectric energy harvester that utilizes a magnetic effect. The static and dynamic behavior of the system was examined using a piecewise function lumped parameter model of a single-degree-of-freedom system with magnetic nonlinearity. The static and frequency variations with separation distance were divided into bistable and monostable regimes by a threshold distance of 9 mm. A nonlinear softening behavior was observed in the monostable and transition region. In contrast, a nonlinear hardening effect dominated in the bistable region. Furthermore, increasing the excitation level induced softening, hardening, and combined behaviors. Combining magnetic nonlinearity with vibro-impact produced a greater bandwidth than utilizing them independently, where a 120% increment was achieved compared to the conventional Vipro-impact energy harvester. Furthermore, a parametric study was explored to optimize the harvester’s performance. In addition, for future work, we are targeting the investigation of the dynamic behavior of the triboelectric energy harvester under random excitations, and testing the harvester under different applications within the ambient range. Magnetic nonlinearity can extend the bandwidth of vibro-impact triboelectric energy harvesters, allowing a more comprehensive range of applications. 

## Figures and Tables

**Table 1:** Physical and geometrical parameters to be used in the model.

Parameters	Symbol	Value
Beam (length × width × thickness)	L×b×h	(75×10×1) mm
Beam Young’s modulus	*E*	69 Gpa
Beam density	ρ	2700 kg/m${}^{3}$
Impact damping coefficient	$c_{i}$	3.2c N.s/m
Impact stiffness coefficient	$k_{i}$	$3.2\;k_{eq}$ N/M
PDMS layer thickness	*T*	$1\times 10^{-3}$ m
Resistance	*R*	10 MΩ
Magnets side length	$L_{m}$	8.0 mm
Magnetic moment	$q_{1}=q_{2}$	0.5 A${}^{2}$/m
PDMS (length × width × thickness)	$L_{p}\times b_{p}\times h_{p}$	(20×20×1) mm
PDMS vacuum permittivity	$\epsilon_{0}$	$8.854\times 10^{-12}$

## Data Availability

Data available on request from the authors.

## Appendix A. The Coefficients of the Expanded Magnetic Force

(A1)
$$\begin{array}{cc} \alpha_{1} & =\frac{F_{R}\left(d^{2}-4Y_{s}^{2}\right)}{{\left(d^{2}+Y_{s}^{2}\right)}^{7/2}}\\ \alpha_{2} & =\frac{5F_{R}Y_{s}\left(-3d^{2}+4Y_{s}^{2}\right)}{2{\left(d^{2}+Y_{s}^{2}\right)}^{9/2}}\\ \alpha_{3} & =\frac{5F_{R}\left(d^{4}-12d^{2}Y_{s}^{2}+8Y_{s}^{4}\right)}{2{\left(d^{2}+Y_{s}^{2}\right)}^{11/2}}\\ \alpha_{4} & =\frac{35F_{R}Y_{s}\left(5d^{4}-20d^{2}Y_{s}^{2}+8Y_{s}^{4}\right)}{8{\left(d^{2}+Y_{s}^{2}\right)}^{13/2}}\\ \alpha_{5} & =\frac{7F_{R}\left(5d^{6}-120d^{4}Y_{s}^{2}+240d^{2}Y_{s}^{4}-64Y_{s}^{6}\right)}{8{\left(d^{2}+Y_{s}^{2}\right)}^{15/2}}\\ \alpha_{6} & =\frac{21F_{R}Y_{S}\left(-35d^{6}+280d^{4}Y_{s}^{2}-336d^{2}Y_{s}^{4}+64Y_{s}^{6}\right)}{16{\left(d^{2}+Y_{s}^{2}\right)}^{17/2}}\\ \alpha_{7} & =\frac{15F_{R}\left(7d^{8}-280d^{6}Y_{s}^{2}+1120d^{4}Y_{s}^{4}-896d^{2}Y_{s}^{6}+128Y_{s}^{8}\right)}{16{\left(d^{2}+Y_{s}^{2}\right)}^{19/2}}\\ \alpha_{8} & =\frac{165F_{R}Y_{s}\left(63d^{8}-840d^{6}Y_{s}^{2}+2016d^{4}Y_{s}^{4}-1152d^{2}Y_{s}^{6}+128Y_{s}^{8}\right)}{128{\left(d^{2}+Y_{s}^{2}\right)}^{21/2}}\\ \alpha_{9} & =\frac{55F_{R}\left(-21d^{10}+1260d^{8}Y_{s}^{2}-8400d^{6}Y_{s}^{4}+13440d^{4}Y_{s}^{6}-5760d^{2}Y_{s}^{8}+512Y_{s}^{10}\right)}{128{\left(d^{2}+Y_{s}^{2}\right)}^{23/2}} \end{array}$$
